# Screening and Evaluation of Dermo-Cosmetic Activities of the Invasive Plant Species *Polygonum cuspidatum*

**DOI:** 10.3390/plants12010083

**Published:** 2022-12-23

**Authors:** Vanille Quinty, Cyril Colas, Rouba Nasreddine, Reine Nehmé, Christine Piot, Micheline Draye, Emilie Destandau, David Da Silva, Gregory Chatel

**Affiliations:** 1 EDYTEM, CNRS, Univ. Savoie Mont Blanc, 73000 Chambéry, France; 2 ICOA, CNRS—UMR 7311 BP 6759, Univ. Orléans, CEDEX 2, 45067 Orléans, France; 3 CBM, CNRS—UPR 4301, Univ. Orléans, CEDEX 2, 45071 Orléans, France

**Keywords:** *Polygonaceae*, *Polygonum cuspidatum*, invasive plant species, superoxide dismutase, hyaluronidase, elastase, collagenase, tyrosinase

## Abstract

*Polygonum cuspidatum* (*P. cuspidatum*) is among the world’s most problematic invasive plant species with negative ecological, socio-economic and security consequences. Management operations in areas invaded systematically generate a large quantity of plant waste, most often without outlets. Using this plant material could constitute a new alternative treatment for sustainable management. *P. cuspidatum* is well known to have numerous biological properties, containing notably stilbenes, quinones, flavonoids and phenolic acids. The present work proposes a reliable strategy using powerful techniques for the screening and the evaluation of the dermo-cosmetic potential of its aerial parts (AP) and root parts (RP). To the best of our knowledge, only antioxidant and anti-tyrosinase activities were previously evaluated on *P. cuspidatum* among the targets studied (superoxide dismutase, hyaluronidase, elastase, collagenase and tyrosinase). The results revealed strong antioxidant and anti-collagenase activities, moderate anti-hyaluronidase activity, while weak anti-elastase and anti-tyrosinase activities were observed for ethanolic extracts. Different standards selected and screened on the same targets made it possible to correlate the observed residual activities of produced extracts of *P. cuspidatum* from Savoie Mont Blanc and their chemical compositions. A structure-activity study was thus conducted on main molecular families, widely represented in the genus *Polygonum*.

## 1. Introduction

Invasive alien species (IAS), including invasive plant species [1], constitute one of the main causes of erosion of biodiversity on a global scale, along with the destruction of natural habitats, the overexploitation of resources, pollution and climate change [2]. Native to the southern and oceanic regions of eastern Asia (Japan, Korea, China), Japanese knotweed, or *Polygonum cuspidatum* Siebold. & Zucc., is an example of a geophyte plant in the family *Polygonaceae*. It was first introduced to Britain as an ornamental plant in the 1840s, not least because of its heart-shaped green leaves, graceful habit and fall bloom [3]. At the beginning of the 20^th^ century after a latency phase, it began its impressive colonisation with a very strong dynamic expansion. A simple fragment of the plant, of a few grams, is enough to create a clone [4]. Thus, *P. cuspidatum* quickly became invasive in Europe, America and Australia [5,6]. It is considered by the International Union for Conservation of Nature (IUCN) as one of the hundred most worrying invasive alien species in the world with negative socio-economic and ecological impacts potentially affecting recipient ecosystems. The species dominates the soil space with its dense and deep root network and with its several meters high, prevents light from passing through, depriving other plant species of the sun. Established on the riverbanks, the good water supply and the richness of the substrate in nutrient elements allows it to have optimal growth and competitiveness, which leads to extensive monospecific stands. This type of stand participates in the collapse of the soil and the erosion of the banks in the alluvial zones, increasing the risks of overflow and flooding [7].

The whole of the French territory is colonised by *P. cuspidatum*, often in wetlands, disturbed lands and areas degraded by anthropic activities [8]. In the French departments of Savoie and Haute-Savoie, it grows in rivers such as the Doron de Beaufort, the Arly or the Chaise in the Beaufortain and Tarentaise valleys [9]. There are several control methods to prevent the spread of the plant [10]. However, the management costs related to the fight against this weed within these territories are substantial and the actions carried out lead to a large quantity of waste most often without outlet [11]. On the Savoie Mont Blanc territory, the chemical and socio-economic valorisation of waste of this high-potential invasive plant species could thus make it possible to reconcile both biodiversity issues and economic development at the local level. Indeed, the specialised metabolites contained in the plant represent a definite opportunity for a circular economy of territory [12,13].

Used in traditional Asian medicine [14] alone or in remedies in combination with other plants for centuries, *P. cuspidatum* can prevent bacterial infections, relieves joint pain, treats inflammations or diabetes. It is widely explored and currently used in nutraceutical products intended to protect the cardiovascular system [15,16]. It was commonly used in China and Japan in daily food [17] and can also be considered a valuation potential as a honey source [18]. It can also serve as a phytoremediator of heavy metals [19]. This plant presents many activities such as antioxidant [20,21,22], antiglycoxidative [23], anti-tyrosinase [24,25,26,27], antimycobacterial, antimicrobial, antidiabetic, anti-inflammatory, anticancer [18,28,29,30], gastroprotective [31], anti-cholinesterase [32] and gingival wound healing [33]. More recently, a study has demonstrated the effectiveness and safety of the plant for acute Respiratory Tract Infections (RTIs) in adults and children [34], and it can also fight the novel severe acute respiratory syndrome coronavirus 2 (SARS-CoV-2) [35]. Numerous bioactive properties of *P. cuspidatum* are due to its various, widely described primary and specialised metabolites from different or specific extraction conditions and plant organs, such as its non-cellulosic polysaccharides [36], its flavonoids, phenolic acids such as chlorogenic acid, stilbenes such as resveratrol and polydatin, anthraquinones such as emodin, volatile constituents, terpenes, naphthalene derivatives [17], proanthocyanidins, carotenoids [18] and even phytosterols and saponins. Moreover, the success of the invasion of this plant and its impact on native populations may be due to the inhibitory effects of its specialised metabolites on plant communities and microorganisms and to the important role of allelopathy [37].

One of the important challenges of the 21st century is the prevention of abiotic stresses at the origin of the aging of the organism. For decades, the fight against the aging of the body, and more particularly that of the skin which is visible, remains an essential point. Skin aging, whether intrinsic or extrinsic, is a complex process resulting from multiple biological, biochemical and physical interactions causing damage that will alter skin functions. Reactive Oxygen Species (ROS) are generated by inducing angiogenesis, however melanogenesis is also activated. Marked degenerative changes then occur in the connective tissue of the upper dermis with degradation of hyaluronic acid, hyperpigmentation or even alterations of elastic fibers and collagen. In keratinocytes, fibroblasts and other inflammatory cells, an increase in the expression of hyaluronidase, elastase, matrix metalloproteinase or collagenase and tyrosinase is observed. Different enzymatic mechanisms are then involved depending on the enzyme considered. Several types of knotweed extracts obtained with various extraction methods or isolated compounds can act as antioxidant agents [21,25,29,32,38,39,40,41,42,43] or skin whiteners [24,25,26,44,45,46,47] and can facilitate treatments for skin diseases [27,48,49,50]. Moreover, resveratrol is now an active ingredient broadly used in cosmetology and dermatology [51,52,53]. To the best of our knowledge, *P. cuspidatum* has only been evaluated for its antioxidant and anti-tyrosinase abilities, among the dermo-cosmetic targets studied (superoxide dismutase, hyaluronidase, elastase, collagenase and also tyrosinase) [21,22,24,25,26,27]. It was in this context that we proposed within the framework of this study the implementation of a strategy allowing the evaluation, with a more global approach, of all the protective activities of our extracts on an exhaustive panel of enzymes involved in the aging of the skin. Moreover, a few representative standards were selected to be screened on enzymatic targets mentioned above and monitored using UHPLC-UV/HRMS/MS structural analysis in produced extracts. The main goal was to correlate the observed activities of the extracts with their phytoconstituents, and subsequently to propose for the first time structure–activity relationships on the main families of specialized metabolites found in the genus *Polygonum*.

## 2. Results and Discussion

### 2.1. Screening and Evaluation of the Dermo-cosmetic Potential of Crude Ethanolic Extracts of Invasive P. cuspidatum 

#### 2.1.1. Determination of Antioxidant Activity of Extracts of AP and RP of *P. cuspidatum*

**Figure 1:**
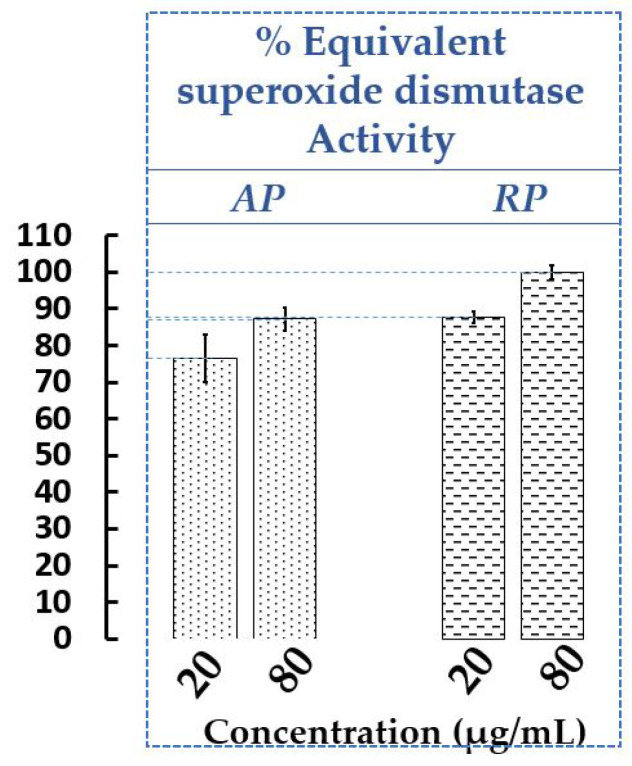
Equivalent SOD activity of AP and RP extracts of *P. cuspidatum* from Savoie Mont Blanc. *n* = 3 independent experiments for each concentration (µg/mL). Bars depict ± S.D.

Free radicals are known to cause oxidative stress affecting the proper functioning of the body and promoting cell aging. Many studies on natural antioxidants from plants have been carried out, in order to find molecules capable of capturing these free radicals and thus preventing damage [54]. Several non-comparable methods are available to evaluate antioxidant capacity involving various mechanisms. It is often recommended to use at least two different methods [55]. In our work, the antioxidant activities of the AP and RP extracts of *P. cuspidatum* from Savoie Mont Blanc were measured both with 2,2-diphenyl-1-picrylhydrazyl radical (DPPH), a widely used and valid method (See Supplementary Materials, Figure S1) and with xanthine oxidase (XOD). XOD is a cytosolic enzyme that catalyzes the metabolizing of hypoxanthine and xanthine to uric acid, and the reduction of O_2_ to superoxide radical O_2_^•−^. XOD associated with a coloured reagent allows us to observe indirectly the protection against ROS using an extract or superoxide dismutase (SOD). SOD is well known as a catalyst in the disproportionation of two superoxide radicals into hydrogen peroxide and oxygen. Therefore, the effect sought was the inhibition of XOD indirectly showing an increase of SOD responsible in vivo for the elimination of O_2_^•−^, thus protecting cells from damage associated with free radicals produced by metabolism. In our case, there is no SOD but the extracts at two concentrations (20 and 80 µg/mL) were tested on XOD (equivalent SOD activity) to evaluate their protective capacities against ROS (Figure 1).

For the chemical and enzymatic assays, the crude ethanolic extracts of AP and RP had a strong antioxidant capacity. With the DPPH assay (See Supplementary Materials, Figure S1), it was the RP extract which has the most significant radical scavenging activity compared to the AP extract for all tested concentrations (for example 86.72 ± 2.48% and 74.06 ± 7.01% at 500 µg/mL, respectively). Of the two lowest concentrations evaluated (5 and 25 μg/mL), the AP and RP extracts have a higher antioxidant power than trolox under the same conditions. These results confirm what was observed previously with the ethanolic extracts of the plant, although the operating protocols may slightly differ [21,40,56]. Indeed, the RP extract was found to be a better antioxidant than the AP one [21]. However, at 500 µg/mL, the RP extracts had percentages of DPPH activity slightly weaker than in our work (i.e., 65–75%), otherwise correlated with their high content of phenols and flavonoids [40,56].

With the enzymatic assay (Figure 1), XOD inhibition was more pronounced for the RP than the AP, with a dose-response effect. Thus, the RP had a slightly higher equivalent SOD capacity than the AP at the same concentration, at 20 µg/mL, for example, 88.76 ± 1.65% and 76.53 ± 6.55%, respectively. From the methanolic extracts of *P. cuspidatum* harvested in Kochi [22], the antioxidant capacity of both the different organs (steam, leaf, rhizome and tuber) of the plant and resveratrol were demonstrated (RP > AP > resveratrol) and confirmed our trend although the extraction solvent was different. Moreover, even if resveratrol is a well-known active stilbene in genus *Polygonum*, its antioxidant contribution was found to be small suggesting that other molecules participate in the overall activity of the extracts [22]. In another study performed in vivo on mice with doxorubicin-induced oxidative stress cardiomyopathy [57], resveratrol and its glycosylated form (polydatin) were shown to increase levels of SOD, catalase and glutathione peroxidase in plasma and significantly reduce the malondialdehyde content in myocardial tissue, a reflection of oxidative stress. Moreover, the in vivo mutual transformation between polydatin and resveratrol maintained the balance, which may then mean that polydatin could replace resveratrol in antioxidants for clinical use.

#### 2.1.2. Determination of the Influence of AP and RP Extracts from Invasive *P. cuspidatum* on Hyaluronidase, Elastase, Collagenase and Tyrosinase Residual Activities 

Skin aging is governed by two main processes [58]. On the one hand, there is a natural or intrinsic, genetically determined process where skin cells enter into senescence. The maintenance of the skin is then altered: the activity of the sebaceous and sweat glands decreases making it less supple and dry, the number of melanocytes decreases causing the appearance of pigmentation irregularities, and the dermis loses elasticity and cell renewal slows down [58]. On the other hand, extrinsic factors such as exposure to UV rays from the sun, atmospheric pollution, nicotine, stress, an unbalanced diet, dehydration and sedentary lifestyle accelerate aging. In addition, the solar UV radiation generates ROS which will both induce angiogenesis but also activate melanogenesis. In keratinocytes, fibroblasts and other inflammatory cells, there is an increase in the expression of hyaluronidase, elastase, matrix metalloproteinase or collagenase and tyrosinase [58]. 

**Figure 2:**
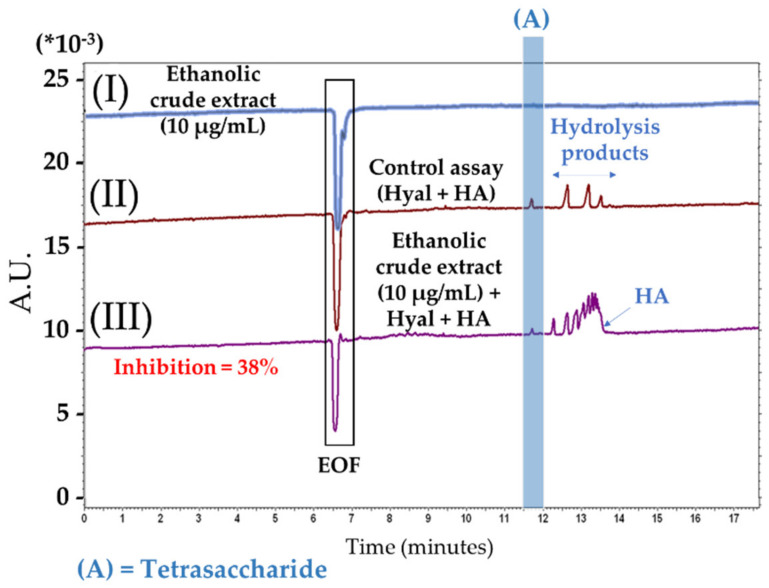
Examples of electropherograms (I−III) showing the inhibitory effect on hyaluronidase of ethanolic crude extract of AP of *P. cuspidatum* from Savoie Mont Blanc. (I) AP extract at 10 µg/mL; (II) Enzymatic assay in absence of AP extract and (III) Enzymatic assay in presence of AP extract at 10 µg/mL. Hyal: hyaluronidase and HA: hyaluronic acid. Reaction mixture in IB of control: 200 µg/mL Hyal and 800 µg/mL of HA. Modulation of hyaluronidase activity experimental conditions: 200 µg/mL of Hyal, 800 µg/mL of HA and 10 µg/mL of filtered crude extract. Peaks identification: electroosmotic flow (EOF) at 6.5 min and peak (A): tetrasaccharide migrating at 11.7 min.

**Figure 3:**
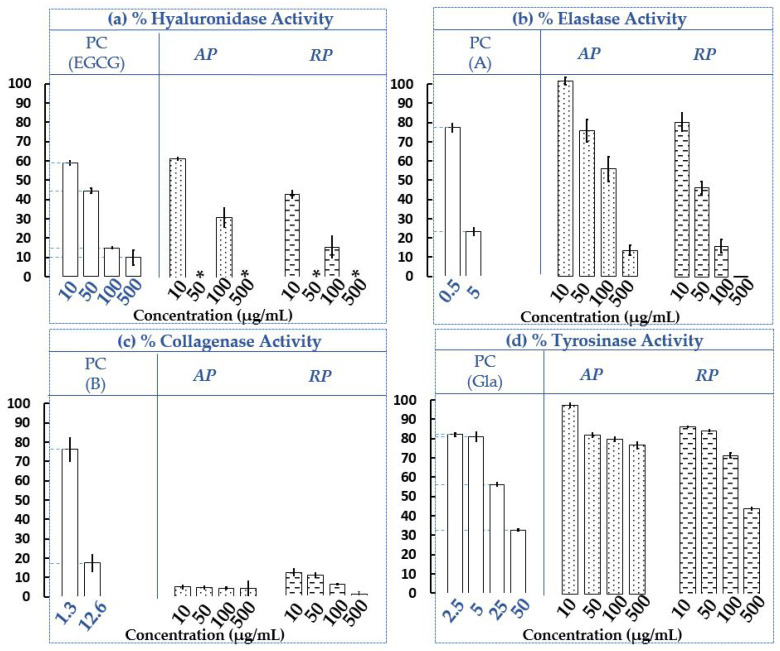
Evaluation of hyaluronidase (**a**), elastase (**b**), collagenase (**c**) and tyrosinase (**d**) residual activities of ethanolic extracts of AP and RP of invasive *P. cuspidatum*. PC = Positive Control; (EGCG) = epigallocatechine gallate; (A) = N-Methoxysuccinyl-Ala-Ala-Pro-Val-chloromethyl ketone; (B) = 1,10-Phenanthroline, monohydrate and (Gla) = glabridin. *n* = 3 independent experiments for each concentration (µg/mL). Bars depict ± S.D. * NT = Not tested.

To miniaturise enzymatic assays, several methods can be implemented, such as 96-wells microtiter plates (MTP) or capillary electrophoresis (CE) [59]. In recent years, CE has received increasing enthusiasm in the determination of active molecules present in complex matrices such as plants [60,61,62,63]. This analytical separative technique allows the direct measurement of the inhibition or activation of the targeted enzyme, following the increase or decrease of the reaction substrate(s) and/or product(s). It requires very small amounts of samples (a few nanoliters) and limits the waste of solvents [61]. Hyaluronidase is one of the endotypic glycosidases that hydrolyzes the endo-N-acetylhexosaminic bonds of hyaluronic acid (HA). Based on the bacterial source of these enzymes, the final reaction products consist mainly of tetrasaccharides [64]; examples of electropherograms obtained in our work with CE-UV are given in Figure 2. The obtained results of the four enzymatic targets (hyaluronidase, elastase, collagenase and tyrosinase) are presented in Figure 3. Moreover, all the tests were carried out under the same experimental conditions as the positive controls (PC), making it possible to validate all the experiments. Four final concentrations of the invasive plant from Savoie Mont Blanc extracts (AP and RP) were evaluated on each enzymatic target, namely 10, 50, 100 and 500 µg/mL. According to our current knowledge, this is the first report where anti-hyaluronidase, anti-elastase and anti-collagenase activities were evaluated on ethanolic extracts of *P. cuspidatum*. 

The results (Figure 3a–d) revealed different efficacies of the extracts on the four different targets. It seems by observing the residual activities of the two extracts on the four enzymes at the lowest concentration, i.e., 10 μg/mL, that the strongest inhibitions observed (from 100 to 0%) are for collagenase (Figure 3c). The anti-collagenase activities of the extracts were thus qualified as strong. Compared to collagenase (also at 10 µg/mL), the anti-hyaluronidase activities can be considered as moderate (Figure 3a), and the anti-elastase and anti-tyrosinase activities as weak (Figure 3b,d). Then considering all the concentrations, it seems that both the AP and RP extracts present strong anti-collagenase activity, moderate anti-hyaluronidase and anti-elastase activities, usually dose dependently. AP and RP seem to have weak anti-tyrosinase activity at all concentrations, except at 500 µg/mL for RP where the activity seems moderate (Figure 3d). Furthermore, it appears that RP exhibits weaker residual activities, on all the targets than AP at the same concentrations, suggesting a stronger inhibitory potential. 

On hyaluronidase (Figure 3a), residual activities of AP and RP at 100 µg/mL for example, are 31.31 ± 4.86% and 15.64 ± 5.43%, respectively, with this last figure comparable to EGCG (PC) at the same concentration, i.e., 14.91 ± 0.49%. Furthermore, the electropherograms (Figure 2) depict the ethanolic crude extract of AP at 10 µg/mL (I), with the enzymatic assay occurring normally in absence of the plant extract (II) and in the presence of 10 µg/mL of the crude extract (III), confirming the absence of interferents with the tetrasaccharide (peak A), the final product of HA hydrolysis. Concentrations at 50 and 500 µg/mL were not tested but with the observation of the PC, it seems that the same trend could be observed. Considering elastase (Figure 3b), the PC(A) is well-known as a strong target inhibitor. The highest concentration tested for the PC(A) was 5 µg/mL, half of the lowest concentration tested for extracts, i.e., 10 µg/mL. It turns out that at 5 μg/mL, the inhibition of the PC(A) is much greater than that observed for the extracts at 10 μg/mL. It is thus assumed that for a doubled concentration of the PC(A), the inhibition would be even higher and the difference between the PC(A) and the extracts even greater. Both extracts (AP and RP) appear to be less potent inhibitors than N-methoxysuccinyl-Ala-Ala-Pro-Val-chloromethyl ketone (A; PC) under the test conditions. 

To have an efficiency similar to that observed at 0.5 µg/mL for PC(B) i.e. 77.42 ± 2.14%, the AP extract at 50 µg/mL (100 times more concentrated compared to 0.5 µg/mL) or the PR extract at 10 µg/mL (20 times more concentrated compared to 0.5 µg/mL) could be used under the assay conditions with respective efficiencies of 76.49 ± 5.95% and 80.42 ± 4.73%. Collagenase (Figure 3c) is strongly inhibited by the AP and RP extracts, with a dose-response effect observed for the RP and a plateau effect for the AP. At 10 µg/mL for example, extracts of the AP and RP seem to be better inhibitors on collagenase than 1,10-Phenanthroline, monohydrate (B; PC), especially since the PC concentration is slightly higher, i.e., 12.6 µg/mL. Indeed, their residual activities on the enzyme are 4.98 ± 0.92%, 12.01 ± 1.65% and 17.49 ± 4.37%, respectively, for the AP, RP and PC. Finally, tyrosinase (Figure 3d) is weakly inhibited by the AP and RP extracts compared to the glabridin (PC) at 50 µg/mL under the assay conditions. The RP extract seems to have a slightly stronger inhibitory potential than the AP extract, at the same evaluated concentrations. For example, at 100 µg/mL, tyrosinase residual activities are respectively 80.21 ± 1.17% and 71.55 ± 1.28% for the RP and AP. Several previous studies had evaluated the potential of Japanese knotweed as a skin whitening agent and support our results [24,25]. Indeed, although in vitro operating conditions were slightly different, previous results showed the dose-response effects, with minor inhibitions at low doses (<50 µg/mL) and moderate efficacies for higher concentrations such as 85.20 ± 1.23% at 100 µg/mL. Tests on the B16-F10 cells also showed the same trend [25]. In another study conducted on the RP extract of *P. cuspidatum*, isolated anthraquinones (physcion, emodin, citreorosein, and anthraglycoside B) were shown to be potential anti-tyrosinase candidates for dermal use [24]. 

For the first time, residual activities of the AP and RP extracts of *P. cuspidatum* on hyaluronidase, elastase and collagenase targets were evaluated. It was found that AP and RP were very good candidates as skin inhibitors of these three enzymatic targets. In contrast to tyrosinase, low to moderate inhibitory effects were observed on the crude ethanolic extracts produced. RP, however, exhibited slightly greater anti-tyrosinase activity than AP. Another screening and evaluation of the dermo-cosmetic potential of the mixture of *P. cuspidatum* parts (50:50; *v*:*v*; AP:RP) were also carried out in order to mimic a whole plant extract (See Supplementary Materials, Figure S2). Of the targets studied (elastase, collagenase and tyrosinase), the mixture (AP:RP) presents similar or slightly higher percentages of inhibition compared to the organs taken independently, suggesting a possible synergy between the two parts. This study thus offers new ways of using the whole invasive plant in the dermo-cosmetic field.

Isolation and purification of biomarkers of interest could however be time-consuming and expensive steps in the analysis of complex matrices such as plant extracts. Thus, we then evaluated the anti-tyrosinase activity on the two organs (AP, RP) *via* the HPTLC-Bioautography/HRMS approach to identify the main biomarkers present in the crude ethanolic extracts. This technique, visual and relatively simple to implement, has made it possible to highlight structure–activity relationships for the two organs of the plant that are not reported in the literature, to the best of our knowledge. It was also the only activity described and compatible with this method among the four enzymes screened (hyaluronidase, elastase, collagenase, tyrosinase) in this work.

### 2.2. Use of the HPTLC-Bioautography/HRMS Approach for the Screening and Identification of Potential Tyrosinase Inhibitors in Ethanolic Extracts of AP and RP of Invasive P. cuspidatum

**Figure 4:**
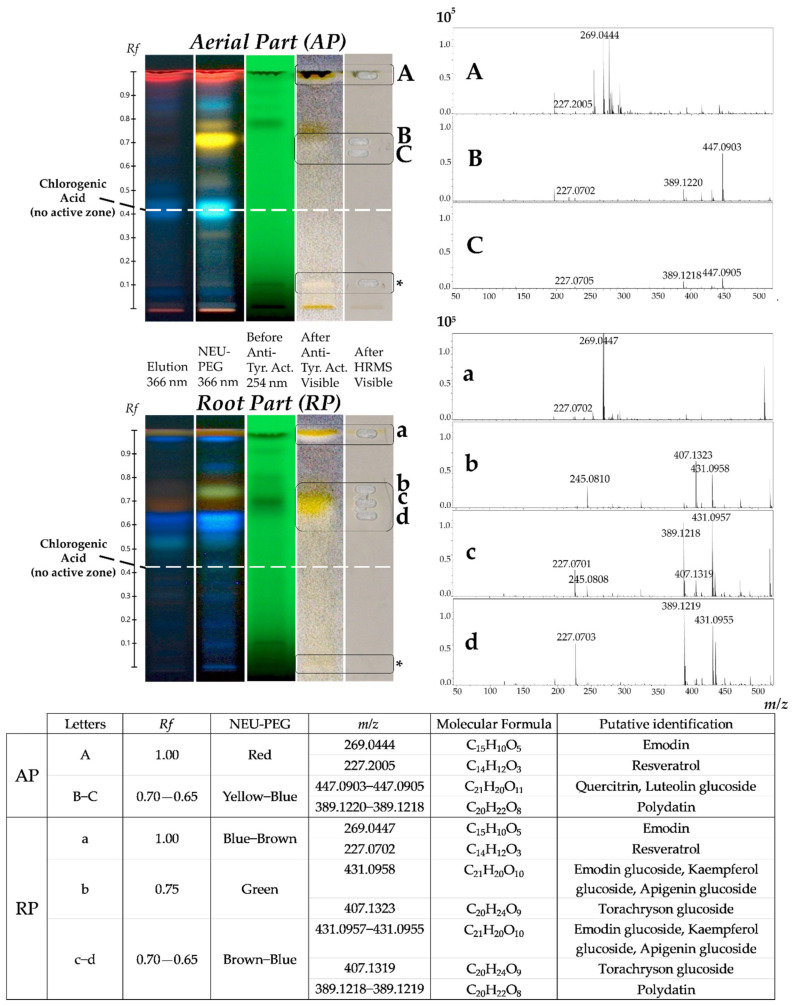
Detection of three active zones in AP and three active zones in RP on tyrosinase (Tyr.) circled in black on the plate. Putative identification of potential tyrosinase inhibitors using HPTLC-Bioautography/HRMS in the negative ionization mode; zones marked with a letter. Mobile phase: EtOAc:FA:AcOH:H_2_O (100:11:11:27) (*v:v:v:v*). Act. = Activity. *Rf* = Retention factor. NEU-PEG: chemical derivatizations NEU then PEG. *m/z*: mass-to-charge ratio. * Not Detected.

Regarding *P. cuspidatum*, several TLC works have been carried out in recent years on the separation, identification and quantification of its various constituents present in its different organs [14,65,66,67,68,69,70]. In our work, a TLC analysis was performed notably to determine anti-tyrosinase activity of invasive plant from Savoie Mont Blanc. Employing this fast and efficient technique to highlight structure–activity and identify biomarkers can be a great help. It is possible to combine, with an appropriate mobile phase, the separation of molecules of the extract (polyphenolic compounds in this work), and certain compatible biological analysis such as tyrosinase [71]. Polyphenolic phytochemical fingerprints of crude ethanolic extracts of AP and RP were evaluated after both chemical and enzymatic revelations. At the end of the NEU-PEG derivation, polyphenolic compounds are detectable at 366 nm with different characteristic colours. It has previously been described that an orange spot may contain quercetin, myricetin or chrysin genin; a green spot a genin of apigenin, naringenin, pinocembrin, kaempferol or kaempferide; a yellow spot luteolin genin; a blue spot may consist of phenolic acids [72]; and a brown spot may represent anthraquinone derivatives [73]. Associated with these colours, the frontal ratios (*Rf*) can indicate whether these genins have a glycosylated group (mono-, di- or tri-). In parallel, the anti-tyrosinase activity is carried out on the TLC plate and observed in white light. The brown background represents the conversion of L-Dopa and the white areas the potential inhibitors of the enzyme [74]. The active anti-tyrosinase markers of interest were then characterized more specifically by coupling the separative TLC with a mass spectrometer, used in Full Scan acquisition mode. The results obtained are shown in Figure 4. 

The results presented in Figure 4 revealed different polyphenolic profiles between the extracts of the AP and RP. Mass spectrometry, associated with the dimensions of polarity and colours, made it possible to give a structural hypotheses of the phenolic compounds present in the extracts. Three anti-tyrosinase active zones in the AP (*Rf* 1.00, 0.70–0.65 diffuse and 0.10) and three anti-tyrosinase active zones in the RP (*Rf* 1.00, 0.75–0.65 very diffuse and 0.02) were represented, with different colours after derivation in NEU-PEG. However, AP seemed to be slightly less active than RP, particularly in the medium-polar and apolar zones at the solvent front (zones marked with a letter).

For the *Rf* 1.00 zones (Figure 4A,a), mass information was obtained corresponding to *m/z* 227.2005, *m/z* 269.0444 ions and to *m/z* 227.0702, *m/z* 269.0447 ions respectively for the AP and RP. In the NEU-PEG, a red spot and a blue-brown spot for the AP and RP were observed, respectively. Resveratrol and emodin could thus be offered on these ions respectively, visually masked by potential chlorophylls in the AP. For the *Rf* 0.70–0.65 diffuse zone in AP (Figure 4B,C), a yellow-blue spot in the NEU-PEG was observed. Mass information was obtained corresponding to the ion *m/z* 447.0903, which may correspond to the quercitrin or luteolin glucoside; and the ion *m/z* 389.1220, with its potential fragment *m/z* 227.0702, relating to polydatin (Figure 4B for example). In RP, the *Rf* 0.75–0.65 very diffuse zone (Figure 4b–d) was divided into two sub-zones: the *Rf* 0.75 zone and the *Rf* 0.70–0.65 zone, with respectively green and brown-blue colours in the NEU-PEG. In the green sub-zone *Rf* 0.75 (Figure 4b) were present the ion *m/z* 407.1323 with its potential fragment *m/z* 245.0810, which may correspond to torachryson glucoside, and *m/z* 431.0958, associated with the structural propositions emodin glycoside, kaempferol glycoside and apigenin glucoside. In the brown *Rf* 0.70 zone (Figure 4c) was present the same mass information, but the torachryson glucoside seemed less intense and the emodin glycoside, kaempferol glycoside and apigenin glucoside seemed more intense than in Figure 4b, confirming the diffusion and co-elution of these compounds. It was also observed that another ion at *m/z* 389.1218, with its potential fragment *m/z* 227.0701, may relate to the proposition of polydatin, comparable in AP at the same *Rf*. Finally, zone *Rf* 0.65 (Figure 4d) polydatin also seemed present with more intense ions (*m/z* 389.1219 and *m/z* 227.0703) than Figure 4c. The ion *m/z* 431.0955 was also observed, less intense than Figure 4c, confirming the great diffusivity of this medium-polar zone. No mass information was obtained for the orange spot (*Rf* 0.10) in AP and the blue spot (*Rf* 0.02) in RP zones in the negative ionization mode (Figure 4, * Not Detected).

The difference in anti-tyrosinase activity between the two extracts (AP and RP), particularly in zones *Rf* 0.65–0.70, could be due to their phytochemical singularity and/or the relative abundance of compounds. Indeed, AP contained quercitrin or luteolin glucoside not represented in RP, and RP contained emodin glycoside or kaempferol glycoside or apigenin glucoside not represented in AP. Present in both parts of the invasive plant, polydatin was less intense in AP than in RP. Moreover, among the structural propositions corresponding to the ion *m/z* 431, they could be emodin derivatives, given that the emodin genin alone seemed to be active.

This bioautographic analysis highlighted main potential tyrosinase inhibitors, namely resveratrol, polydatin, emodin and its glucoside derivatives, quercitrin or luteolin glucoside and torachrysone glucoside. A few representative standards of specialized metabolites were then selected to be screened on the four studied enzymatic targets (hyaluronidase, elastase, collagenase and tyrosinase) and monitored in the crude ethanolic extracts (AP and RP) of *P. cuspidatum* using UHPLC-UV-Q-TOF-HRMS/MS. The objective was to correlate the obtained effectiveness of the extracts on targets (Section 2.1.2) with their chemical compositions. Among those mentioned above, we selected the two stilbenes (resveratrol and polydatin) and the quinone (emodin) present both in AP and RP and previously described in studies conducted in vitro and in vivo enzymatic assays [24,25,27,75]. Another compound more present in AP than RP (chlorogenic acid) was also selected, although potentially not active on tyrosinase (See *Rf* 0.42, Figure 4). Indeed, this phenolic acid widely represented in the plant kingdom, has made it possible to expand to other molecular families of the genus *Polygonum* and was known to be a potential candidate in the treatment of skin diseases [76,77,78,79].

### 2.3. Determination of Hyaluronidase, Elastase, Collagenase and Tyrosinase Residual Activities in The Presence of the Four Selected Standards (Resveratrol, Polydatin, Emodin and Chlorogenic acid)

**Figure 5:**
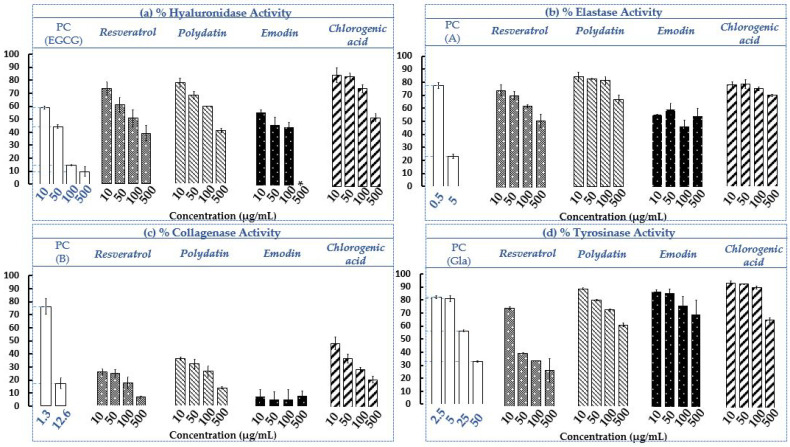
Evaluation of hyaluronidase (**a**), elastase (**b**), collagenase (**c**) and tyrosinase (**d**) residual activities of the four selected standards: resveratrol, polydatin, emodin and chlorogenic acid. PC = Positive Control; (EGCG) = epigallocatechine gallate; (A) = N-Methoxysuccinyl-Ala-Ala-Pro-Val-chloromethyl ketone; (B) = 1,10-Phenanthroline, monohydrate and (Gla) = glabridin. *n* = 3 independent experiments for each concentration (µg/mL). Bars depict ± S.D. * NT = Not tested.

The screening and evaluation of the four selected standards solubilized in EtOH was conducted, under the same experimental conditions used on the AP and RP extracts (See Section 2.1.2). The results are shown in Figure 5 and an example of electropherograms for the control, chlorogenic acid and resveratrol obtained for hyaluronidase assays with CE-UV are given (See Supplementary Materials, Figure S3).

The results presented in Figure 5a–d revealed the different efficacies of the standards on the four different targets. It seems by observing the residual activities of the standards on the four enzymes at the lowest concentration, i.e., 10 μg/mL, that the strongest inhibitions observed (from 100 to 0%) concern the collagenase (Figure 5c). The anti-collagenase activities of the extracts were thus qualified as strong. Compared to the collagenase (also at 10 µg/mL), the anti-hyaluronidase and anti-elastase activities can be considered as moderate (Figure 5a,b), and the anti-tyrosinase as weak (Figure 5d). Then considering all the concentrations, it seems that the four selected standards have moderate capacities to inhibit hyaluronidase and elastase and a strong capacity to inhibit the collagenase (emodin >> resveratrol > polydatin > chlorogenic acid), with low to moderate activities on tyrosinase (resveratrol >> others standards). It was the same trend on the crude extracts of AP and RP of invasive *P. cuspidatum* (Section 2.1.2, Figure 3). Standards show dose-response effects on all dermo-cosmetic targets except for emodin, which on elastase and collagenase presents a plateau effect (error bars ± SD crossing each other). For the two stilbenes, it generally turns out that resveratrol inhibits slightly more than polydatin, its glucosyl, on the four targets at the same concentrations. The sugar group added to the genin moiety may have a slight impact on the inhibitory activity. 

Emodin seems to have the best inhibitory potential on hyaluronidase, on each of the evaluated concentrations (Figure 5a). At 50 µg/mL for example, emodin has a hyaluronidase residual activity of 46.64 ± 5.92% close to PC (44.40 ± 1.30%), followed by resveratrol, polydatin and chlorogenic acid, with 60.57 ± 6.04%, 67.80 ± 2.90% and 84.26 ± 3.13%, respectively. However, at 500 µg/mL, it could not be tested because it was not soluble in the medium at this concentration. In a previous study conducted on *Aloe vera* peel extract, it was found that *aloe*-emodin isolated from the plant could inhibit hyaluronidase by 50% with a concentration of 40 µg/mL [80]; this confirmed the results obtained in our study with emodin. It was shown that chlorogenic acid, isolated from the bark extract of *Ficus microcarpa L. fil.*, exhibited weak inhibition of hyaluronidase (activity of 86.5% at 177.16 µg/mL) [81] while in our study, chlorogenic acid had a better inhibition at a lower concentration of 100 µg/mL (residual activity of 75.40 ± 3.10%). This difference could be explained by the fact that operating conditions were slightly different. In the molecular docking study of chlorogenic acid as a potential hyaluronidase inhibitor, it was demonstrated that hydrogen bonds could be formed between phenolic acid and the hyaluronidase amino acid residues Glu477 and Glu582, and that there could be spatial hydrophobic interactions between the phenyl group of chlorogenic acid and the amino acid residues Ala407 and Val411 [76].

Compared to the three other standards, emodin has the strongest inhibitory potential on elastase (emodin > resveratrol > chlorogenic acid > polydatin), with a plateau effect of ca. 50% of elastase residual activity on all concentrations (Figure 5b). At 10 µg/mL for example, the elastase residual activities of emodin, resveratrol, chlorogenic acid and polydatin are 56.01 ± 0.10%, 74.63 ± 4.29%, 78.15 ± 2.21% and 84.37 ± 3.73%, respectively. It seems that the PC (A) is a stronger inhibitor than the four standards in operating conditions, since for a two times lower concentration, i.e., 5 µg/mL, it has an elastase residual activity of 23.18 ± 1.83%. Several anthraquinones, including emodin and *aloe*-emodin, have been shown to be strong inhibitors of Human Leukocyte Elastase and Cathepsin, two serine proteinases implicated in diseases characterised by the abnormal degradation of connective tissue, such as pulmonary emphysema and rheumatoid arthritis. They had elastase residual activities of 45% and 79%, respectively, probably due to hydrophobic interactions with the enzyme [82]. On human polymorphonuclear leukocyte function, resveratrol also showed interesting elastase inhibition [83,84]. 

On collagenase (Figure 5c), emodin seems to be the strongest inhibitor, exceeding even the PC (B) in operating conditions with collagenase residual activities of 7.33 ± 5.36% and 17.51 ± 4.37% at 10 µg/mL, respectively, especially since the inhibitor (B) concentration is slightly higher, i.e., 12.6 µg/mL. In vitro evaluation and comparison of inhibitory effects of 44 anthraquinones on the bacterial collagenase, emodin was found to be the most potent, capable of inhibiting the enzyme target of 50% with a concentration of only 10.81 µg/mL [85]. The difference with our study could be explained by the operating protocols which are different. Resveratrol has been shown to serve as an agent for the treatment of multiple myeloma with matrix metalloproteinase inhibitory activity [86]. Pure *E*-polydatin was also a very strong inhibitor, with a collagenase residual activity of approximately 25% at 117.12 µg/mL [87] (for 26.43 ± 4.29% in our work). Although chlorogenic acid has the lowest potential compared to other standards and the PC (B), it is still a strong inhibitor with 48.70 ± 4.77% collagenase residual activity at 10 µg/mL. By inhibiting matrix metalloproteinase 2 and 9, chlorogenic acid showed it could ameliorate brain damage and sensory-motor functional deficits [77].

Resveratrol seems to be the strongest tyrosinase inhibitor at all the concentrations tested, with the tyrosinase residual activity ranging from 75.42 ± 1.04% at 10 μg/mL to 27.70 ± 9.10% at 500 μg/mL (Figure 5b). The results on this target are often in contrast to the literature [24,27,87], often depending on the implemented protocol and the type of enzyme used. Indeed, in a previous study on isolated compounds from the root extract of *P. cuspidatum* from Taiwan, stilbenes (resveratrol, polydatin) did not show any residual activities, while the anthraquinones (physcion, emodin, citreorosein, anthraglycoside B) showed moderate to strong inhibition effects, in a cell-free tyrosinase test. Physcion was the most powerful inhibitor (30% of tyrosinase residual activity), also showing significantly higher permeation than emodin [24]. Resveratrol had a much weaker effect on mushroom tyrosinase than on human tyrosinase [75]. Recently, however, it was shown that in melanocytes, polydatin isolated from the plant inhibited tyrosinase and melanin production. Its effects on tyrosinase-related proteins 1 and 2, as well as on a transcriptional factor involved in melanogenesis, were also explored. Polydatin was found to suppress the mRNA and protein expression of enzymes and the transcription factor in a concentration-dependent manner [27]. Pure *E*-polydatin also showed approximately 62% at 117.12 µg/mL of tyrosinase residual activity [87], which was relatively close to our results (73.69 ± 0.99% at 100 µg/mL), although the experimental protocols differed slightly. 

Considering these results on the one hand, selected standards (resveratrol, polydatin, emodin and chlorogenic acid), separately evaluated, participate in anti-hyaluronidase, anti-elastase, anti-collagenase and anti-tyrosinase activities of the extracts (See Section 2.1.2). On the other hand, if this confirmed part of the obtained results on tyrosinase with a HPTLC-bioautographic approach (See Section 2.2), no active zone corresponding to chlorogenic acid in AP and RP was detected (See *Rf* 0.42, Figure 4). It could be that the minimum detectable amount of this compound with this approach was reached (LOD) [88]. It could also be that there are possible synergies between these four different molecules or with other extracted molecules also present in the extracts of invasive *P. cuspidatum* from Savoie Mont Blanc. It was in this context that the four selected standards were monitored using UHPLC-UV/HRMS/MS structural analysis in the AP and RP extracts.

### 2.4. Evaluation of the Chemical Compositions of AP and RP of Invasive P. cuspidatum

**Figure 6:**
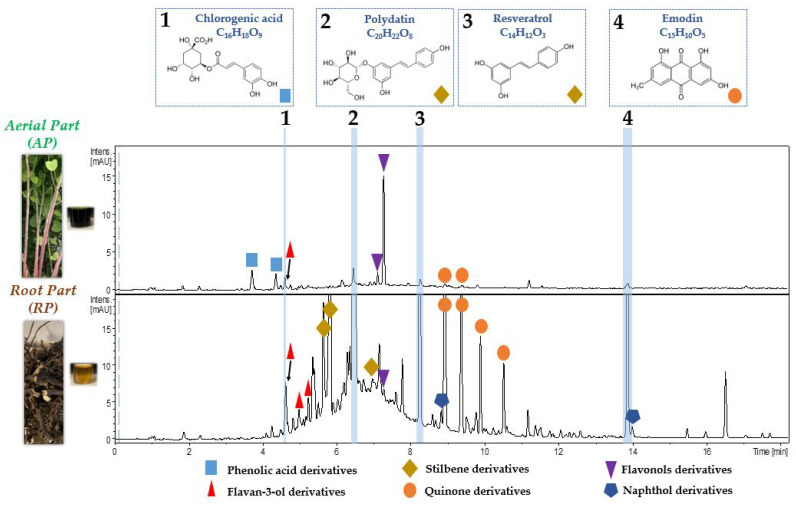
UV Chromatographic fingerprint analysis at 285 nm of AP and RP of invasive *P. cuspidatum* using UHPLC-UV-Q-TOF-HRMS/MS. Identifications of the four specialized metabolites of interest on the four enzymatic studied targets (hyaluronidase, elastase, collagenase and tyrosinase): chlorogenic acid (1), polydatin (2), resveratrol (3) and emodin (4). Other identified compounds corresponding to the derivatives were reported in Supplementary Materials, Table S1.

A liquid chromatography analysis coupled with both UV and mass spectrometry detections was carried out in order to evaluate the chemical compositions of the AP and RP extracts of *P. cuspidatum* present in the Savoie Mont Blanc territory. UV chromatograms obtained at 285 nm are presented in Figure 6.

Comparison of the UV chromatograms made it possible to highlight the difference in chemical compositions between AP and RP of the invasive plant. It was also possible to observe differences in the relative proportions of the main molecules. In the crude AP and RP extracts, 11 and 17 main specialized metabolites (Figure 6) were identified, respectively, in the negative ionization mode. They were notably the phenolic acids with chlorogenic acid, flavan-3-ols with catechin, stilbenes with polydatin and resveratrol, quinones with emodin, flavonols with quercitrin and naphthol derivatives with torachrysone glucoside (See Supplementary Materials, Table S1). This UHPLC-UV-Q-TOF-HRMS/MS analysis made it possible to confirm the presence and the structures of potential anti-tyrosinase biomarkers, identified using HPTLC/HRMS analysis (Section 2.2, Figure 4). For the four compounds monitored (Figure 6, 1–4), numbered from most polar to least polar, depending on the gradient used, their distribution within the invasive plant was different. Indeed, chlorogenic acid (Figure 6, 1) was more abundant in AP than in RP and polydatin, and resveratrol and emodin (Figure 6, 2–4) were much more present in RP than in AP. It was also found in a previous study that the leaves of *P. cuspidatum* were a better source of phenolic acids, flavones and flavonols than the stems or roots of the plant. For them, the roots were also an excellent source of flavan-3-ols (monomers and oligomers) and stilbenes, such as resveratrol and derivatives [41].

Furthermore, these results could explain the differences in residual activities observed on the enzymatic targets examined between the AP and RP extracts (Section 2.1.2, Figure 3 and Section 2.2, Figure 4). RP, which was often slightly more active than AP at the same concentrations, contained more stilbene and quinone derivatives than AP. The compound that seemed to predominate in AP was identified as quercitrin and not luteolin glucoside, as previously suggested (Section 2.2, Figure 4), with its ion fragment at *m/z* 301 and not *m/z* 285 for the aglycones. It could be that this flavonol is mainly responsible for the important residual activities observed on the enzymatic targets tested relatively close to the RP. This does not exclude a possible synergy with the other minority molecules of the extract. The pronounced yellow colour observed in AP on the HPTLC plate (Section 2.2, Figure 4B,C) could otherwise mask a part of the activity in this polarity range. According to the literature, and the description of the phytochemical composition of *P. cuspidatum*, it has previously been shown that various individual flavonoids could inhibit hyaluronidase activity, including condensed tannin, kaempferol, myricetin, quercetin and derivatives such as quercitrin, catechin and epicatechin. Procyanidins have also been shown to have anti-hyaluronidase activity as well as anti-collagenase and some anti-elastase activities [81,89,90,91].

Numerous literature data on phytochemical compositions of species of the genus *Polygonum* were available and confirmed the obtained results [41,92,93,94,95,96]. In this previous work [95], a total of 71 compounds (including phenolic acids, flavan-3-ols, stilbenes, flavonols and flavones, chlorophylls, carotenoids and triterpenoids) were identified in methanolic leaves and rhizomes extracts of *P. cuspidatum* and *P. sachalinensis* from Poland using UHPLC-UV-Q-TOF-HRMS in negative ionization mode. However, chlorogenic acid, resveratrol, polydatin and emodin were more abundant in the leaves than in the rhizomes. This difference with our study could be explained by the fact that the extraction conditions and/or the plant material were slightly different. Indeed, the authors had worked on the leaves of the plant whereas in our case, the leaves are mixed with the stems constituting the aerial part (AP). Furthermore, using the online UHPLC-ABTS technique to evaluate antioxidant activity [95], the authors showed that catechin, procyanidin dimer B, chlorogenic acid, 3-*O*-coumaroylquinic acid and resveratroloside (also identified in our work: see Supplementary Materials, Table S1) were shown to possess the strongest radical scavenging capacity, which could confirm the results obtained and presented above in Section 2.1.1, since they were common compounds. 

## 3. Materials and Methods 

### 3.1. Reagents and Materials

Ethanol used for ultrasound assisted plant extraction (99.5%, HPLC Baker Analyzed, J.T. Baker), acetonitrile (ACN, 99.9%, LC-MS Chromasolv, Honeywell), ethyl acetate (EtOAc, 99.9%), formic acid (FA, ≥99%, LC-MS Optima, Fisher Chemical) and water (LC-MS Chromasolv, Honeywell) used for UHPLC/HRMS and HPTLC/HRMS analysis were purchased from Thermo Fisher Scientific (Illkirch-Graffenstaden, France). The solvents used for biological analysis and HPTLC were ethanol (≥99.8%), methanol (≥99.8%), dimethylsulfoxyde (DMSO, 99%), glacial acetic acid (AcOH), ammonium hydroxyde (28%) and orthophosphoric acid (85%). Acids and bases were HPLC grade and purchased from VWR (Fontenay-sous-Bois, France). EnzChek Elastase Assay Kit (E-12056) and EnzChek Gelatinase/Collagenase Assay Kit (E-12055) were purchased from Thermo Fischer Scientific (Artenay, France). SOD Assay Kit (19160-1KT-F), hyaluronidase type I-S from bovine testes (Hyal, 400–1000 units/mg solid) and tyrosinase from mushroom (Tyr, 8500 units/mg solid), HPTLC aluminium sheets silica gel 60 F_254_ (20 cm × 20 cm, cut in half), diphenylboryloxyethylamine (NEU) prepared in MeOH, polyethyleneglycol 4000 (PEG-4000) prepared in EtOH, polyethyleneglycol 10,000 (PEG-10,000) prepared in phosphate buffer, pellets of sodium hydroxide, 2,2-diphenyl 1-picrylhydrazyle (DPPH, ≥90%), 3,4-dihydro-6-hydroxy-2,5,7,8-tétraméthyl-2H-1-benzopyran-2-carboxylic acid (Trolox, ≥98%), ammonium acetate (AcNH_4_, 98%), sodium acetate (≥99%), 3,4-dihydroxy-L-phenylalanine (L-Dopa, ≥98%, TLC), N-cyclohexyl-3-aminopropanesulfonic acid (CAPS, ≥99%), 3,4′,5-trihydroxy-trans-stilbene, 5-[(1E)-2-(4-hydroxyphenyl)ethenyl]-1,3-benzenediol (resveratrol, ≥99%), resveratrol 3-*O*-*β*-*D*-glucopyranoside (polydatin, ≥95%), 1,3,8-tri hydroxy-6-methyl anthraquinone (emodin, ≥90%), 3-(3,4-dihydroxycinnamoyl)-quinic acid (chlorogenic acid, ≥95%), 4-[(3R)-3,4-dihydro-8,8-dimethyl-2H,8H-(benzo[1,2-b:3,4-b′]dipyran-3-yl)-1,3-benzenediol (glabridin, ≥98%), epigallocatechin gallate (EGCG, ≥95%), Hyaluronic Acid (HA), sodium salt, Streptococcus pyrogenes-CAS 9067-32-7-Calbiochem were purchased from Merck (Molsheim, France). Ultra-pure water was produced with a PureLabFlex system from Veolia (Wissous, France).

### 3.2. Plant Material

The local invasive alien plant *P. cuspidatum* was collected in the municipality of Voglans (Savoie, France) at an altitude of 250 m, on an area of 2 m^2^. The collection was carried out before flowering in July. Its height was about 3 m. Biodiversity conservation actors of department of Savoie have botanically identified wild *P. cuspidatum* on the invaded area. Its AP and RP were dried for 48 h in the Salvis TSW-120-ED oven at 50 °C. The dry matter was then ground and sieved to 0.5 mm using a FRITSCH PULVERISETTE 19 universal knife mill.

### 3.3. Ultrasound Assisted Plant Extraction

The powders of the AP and RP of *P. cuspidatum* (0.5 g) were extracted in Pyrex tubes using a 35 kHz low-frequency ultrasonic bath (BANDELIN Sonorex Super RK 510H) with 5 mL of EtOH for 1 h at 60 °C. Each sample was made in triplicate. The extracts were then filtered through a 0.45 µm D.25 mm PTFE filter. They were then combined and evaporated to dryness under nitrogen to form a single sample (AP or RP) taken up in the extraction solvent at 10,000 μg/mL. Each sample obtained was then diluted in cascade to obtain solutions at 5000; 2000; 1000; 500; 250; 200 and 100 µg/mL used for chemical and enzymatic assays. Initial concentrations (10,000; 2000; 1000 and 200 µg/mL) were used for hyaluronidase, elastase, collagenase and tyrosinase assays. Initial concentrations (1000 and 250 µg/mL) were used for equivalent superoxide dismutase assay. Initial concentrations (10,000; 5000; 1000; 500 and 100 µg/mL) were used for DPPH assay (See Supplementary Materials).

### 3.4. Assays of Enzymatic Activities

Each enzyme having specificities, different powerful techniques and methodologies was implemented in our work. Colorimetric and fluorescent enzymatic activity assays using MTP were conducted to evaluate antioxidant, anti-elastase, anti-collagenase and anti-tyrosinase activities of AP and RP extracts obtained through ultrasound-assisted plant extraction. Anti-hyaluronidase activity was determined using CE allowing a direct measurement of the effect of hyaluronidase which simultaneously catalyzes the degradation and elongation of its substrate [64].

#### 3.4.1. Equivalent Superoxide Dismutase Activity Assay

The determination of SOD activity is carried out by an indirect assay based on XOD and a coloured reagent, using a commercial kit. The assay was based on its ability to inhibit the superoxide anion free radical of O_2_^•−^ generated using the xanthine-xanthine oxidase system. The kit contains a buffer solution and its dilution, a Dojindo’s highly water-soluble tetrazolium salt, a working substrate solution (WST-1; 2-(4-Iodophenyl)-3-(4-nitrophenyl)-5-(2,4-disulfophenyl)-2H-tetrazolium monosodium salt) that produces a water-soluble formazan dye upon reduction with a superoxide anion diluted in buffer solution and a XOD solution prepared in dilution buffer. Reaction buffer and EtOH were used as negative control. In each well, 20 μL of extract or negative control were mixed with 20 μL of XOD solution and 200 µL of WST-1. The plate was incubated for 20 min at room temperature (RT) then agitated. The absorbance (Abs) reading (λ = 450 nm) was performed using a CLARIOstar Plus microplate reader (BMG Labtech, Champigny-sur-Marne, France). Experiments were performed in triplicate. The percentage of equivalent SOD activity was calculated using the following equation:

$$\mathrm{Activity}\;\left(\%\right)=\left(\frac{\left(\mathrm{Abs}\;\mathrm{Ext}-\mathrm{Abs}\;\mathrm{ExtB}\right)}{\left(\mathrm{Abs}\;\mathrm{PC}-\mathrm{Abs}\;\mathrm{CB}\right)}\right)*100$$

where Abs PC (Positive Control) is the absorbance of Buffer with 5% EtOH, XOD and WST-1; Abs CB (Control Blank) is the absorbance of Buffer with 5% EtOH and WST-1; Abs Ext (Extract) is the absorbance of the tested invasive plant extract in the buffer, XOD, and WST-1 and Abs ExtB (Extract Blank) is the absorbance of the tested invasive plant extract in the buffer and WST-1.

#### 3.4.2. Hyaluronidase Activity Assay and Capillary Electrophoresis Conditions

The effects of *P. cuspidatum* extracts and four analytical standards (polydatin, emodin, resveratrol and chlorogenic acid) were evaluated towards hyaluronidase catalytic activity using an offline CE-based assay optimized in a previous study [97]. CE assays were carried out with some adjustment considering the presence of EtOH in plant extracts as well as in standards and its concentration was limited to 5% of the final volume. Briefly, 37.5 µL of incubation buffer were preincubated with 5 µL of hyaluronic acid, HA (at 4000 µg/mL) and 2.5 µL of extracts at 37 °C for 10 min. Then 5 µL of enzyme (at 2000 µg/mL) were added into the mixture. Reactions were incubated for 180 min at 37 °C, then they were stopped by increasing the temperature to 90 °C for 10 min using a hot water bath. Results were compared to those obtained with a referenced inhibitor of hyaluronidase, the EGCG at the same final concentration in the mixture. Control assays, where HA hydrolysis occurred normally in the absence of aerial and root parts extracts or standards, were performed and stopped using the same protocol by substituting the extract volume with 2.5 µL of EtOH. Experiments were performed in triplicate.

Buffers and stock solutions for hyaluronidase CE assay: the incubation buffer (IB) was 2000 µM sodium acetate in deionized water (equivalent to 164 µg/mL) at pH 4.3 (adjusted with 1 M glacial acetic acid). The background electrolyte (BGE) was 50,000 µM ammonium acetate in deionized water (equivalent to 3854 µg/mL) at pH 8.9 (adjusted with 1 M ammonium hydroxyde). All buffers were prepared daily and filtered using hydrophilic PVDF syringe filters before use. HA, BTH, and tetrasaccharide stock solutions were prepared at 10,000 µg/mL in IB and then diluted to the appropriate concentrations. Aliquots of 2000 µg/mL were stored at −20 °C. EGCG was prepared in 100% EtOH at 1000 µg/mL. Extract stock solutions were prepared at different concentrations in 100% EtOH. Buffers, extract stock solutions and standard solutions were all stored at 4 °C.

The analysis of enzymatic reaction solutions as well as raw ethanol extracts were performed using the PA800+ CE apparatus equipped with a photodiode array detector and Beckman 32 Karat software (Sciex, Redwood City, CA, USA). Fused silica capillaries (from Polymicro Technologies, Phoenix, AZ, USA) were used with a total length of 0.57 m (0.47 m effective length) and 50 μm inner diameter. Detection wavelength was set to 200 nm. The capillary was flushed with NaOH 1 M (for 5 min), water (for 0.5 min) and BGE (for 3 min) to ensure a good cleaning of the inner capillary surface between runs. All rinse cycles were carried out at 50 psi and injections (14 nL) were performed using hydrodynamic injections from the anodic side of the capillary at 1.5 psi for 5 s. The separation of the HA degradation products was ensured in positive polarity mode at +15 kV (at 25 °C). The corrected peak area (CPA), determined by the ratio of area to the migration time, was a reliable mean for the tetrasaccharide quantification (final product of HA hydrolysis) after its separation under the electric field inside the capillary. It was followed to assess the enzyme’s activity in the presence of plant extracts and compared to reactions carried out in absence of extracts or standards. The percentage of hyaluronidase activity was calculated using the following equation:

$$\mathrm{Activity}\;\left(\%\right)=\left(\frac{A_{x}}{A_{0}}\right)*100$$

where A_x_ and A_0_ are the CPAs of tetrasaccharide formed in the presence and in the absence of a potential inhibitor (invasive plant extract or standard), respectively.

#### 3.4.3. Elastase Activity Assay

The kit contains a soluble DQ elastin from bovine neck ligament, BODIPY FL conjugate, 1 mg enzyme substrate lyophilized from 1 mL phosphate-buffered saline at pH 7.2 (PBS); a 10× reaction buffer of 1 M Tris-HCl, pH 8.0, containing 2000 µM sodium azide; elastase from pig pancreas, 50 units, where one unit is defined as the amount of enzyme necessary to solubilize 1 mg of elastin in 20 min (pH 8.8, 37 °C); and inhibitor of elastase named N-methoxysuccinyl-Ala-Ala-Pro-Val-chloromethyl ketone. This selective inhibitor of elastase was diluted in 1× reaction buffer prepared from 10× buffer to obtain two solutions at 2.51 and 25.15 μg/mL. Reaction buffer 1× and EtOH were used as negative control. In each well, 10 μL of extract, inhibitor or analytical standards (mentioned above in Section 3.4.2) or negative control were mixed with 40 μL of 1× buffer and 100 μL of elastase (at 0.5 U/mL). The plate was incubated for 5 min at RT then agitated. Then, 50 µL of enzyme substrate at 100 μg/mL were added. The plate was incubated for 2 h at RT in the dark. Finally, fluorescence (Fluo, λ_exc_ 477 nm − λ_em_ 525 nm) was measured using a CLARIOstar Plus microplate reader. Experiments were performed in triplicate. The percentage of elastase activity was calculated using the following equation:

$$\mathrm{Activity}\;\left(\%\right)=\left(\frac{\left(\mathrm{Fluo}\;\mathrm{Ext}-\mathrm{Fluo}\;\mathrm{ExtB}\right)}{\left(\mathrm{Fluo}\;\mathrm{PC}-\mathrm{Fluo}\;\mathrm{CB}\right)}\right)*100$$

where Fluo PC (Positive Control) is the fluorescence of PBS Buffer with 5% EtOH, elastase and enzyme substrate; Fluo CB (Control Blank) is the fluorescence of PBS Buffer with 5% EtOH and enzyme substrate; Fluo Ext (Extract) is the fluorescence of the tested invasive plant extract in the PBS buffer, elastase, enzyme substrate and Fluo ExtB (Extract Blank) is the fluorescence of the tested invasive plant extract in the PBS buffer and enzyme substrate.

#### 3.4.4. Collagenase Activity Assay

The kit contains a soluble DQ gelatin from pig skin, fluorescein conjugate, 1 mg enzyme substrate lyophilized from 1 mL phosphate-buffered saline at pH 7.2 (PBS); a 10× Reaction Buffer of 0.5 M Tris-HCl, 1.5 M NaCl, 50,000 µM CaCl_2_ (equivalent to 5549 µg/mL), pH 7.6, containing 2000 µM sodium azide; collagenase, Type IV from *Clostridium histolyticum*, 500 units, where one unit is defined as the amount of enzyme required to liberate 1 µmole of L-Leucine equivalent from collagen in 5 h (pH 7.5, 37 °C); and inhibitor of collagenase named 1,10-Phenanthroline, monohydrate. This general metalloproteinase inhibitor of collagenase was diluted in 1× reaction buffer prepared from 10× buffer to obtain two solutions at 3.60 and 36.04 μg/mL. Reaction buffers 1× and EtOH were used as negative control. In each well, 10 μL of extract, inhibitor or analytical standards (mentioned above in Section 3.4.2) or negative control were mixed with 70 μL of 1× buffer and 100 μL of collagenase (at 0.4 U/mL). The plate was incubated for 5 min at RT. Then, 20 µL of enzyme substrate at 125 μg/mL were added. The plate was incubated for 2 h at RT in the dark then agitated. Finally, fluorescence (Fluo, λ_excitation_ = 483 nm − λ_emission_ = 530 nm) was measured using a CLARIOstar Plus microplate reader. Experiments were performed in triplicate. The percentage of collagenase activity was calculated using the following equation:

$$\mathrm{Activity}\;\left(\%\right)=\left(\frac{\left(\mathrm{Fluo}\;\mathrm{Ext}-\mathrm{Fluo}\;\mathrm{ExtB}\right)}{\left(\mathrm{Fluo}\;\mathrm{PC}-\mathrm{Fluo}\;\mathrm{CB}\right)}\right)*100$$

where Fluo PC (Positive Control) is the fluorescence of PBS Buffer with 5% EtOH, collagenase, and enzyme substrate; Fluo CB (Control Blank) is the fluorescence of PBS Buffer with 5% EtOH and enzyme substrate; Fluo Ext (Extract) is the fluorescence of the tested invasive plant extract in the PBS buffer, collagenase, enzyme substrate and Fluo ExtB (Extract Blank) is the fluorescence of the tested invasive plant extract in the PBS buffer and enzyme substrate.

#### 3.4.5. Tyrosinase Activity Assay

Tyrosinase inhibitory activity was determined using a previously described protocol with slight modifications [98]. The phosphate buffer solution (PBS at 10,000 µM, pH = 6.8) was prepared by mixing 2 M NaOH, 1 M H_3_PO_4_ and ultrapure water in the proportions (1:1.45:250). Glabridin was prepared in DMSO at 10,000 µM (equivalent to 3243 µg/mL), then diluted in EtOH to obtain solutions at 16.22; 32.44; 162.19 and 324.38 µg/mL and was used as a positive control. PBS and EtOH were used as negative control. In each well, 10 μL of extract, inhibitor or analytical standards (mentioned above in 3.4.2.) or negative control were mixed with 30 μL of PBS and 40 μL of tyrosinase solution (at 125 U/mL). The plate was incubated for 5 min at RT. Then, 120 μL of 8300 µM (equivalent to 1636 µg/mL) L-Dopa substrate solution were added to the mixture and the plate was incubated 30 min at RT in the dark then agitated. The absorbance (Abs) reading (λ = 490 nm) was performed using a CLARIOstar Plus microplate reader. Experiments were performed in triplicate. The percentage of tyrosinase activity was calculated using the following equation:

$$\mathrm{Activity}\;\left(\%\right)=\left(\frac{\left(\mathrm{Abs}\;\mathrm{Ext}-\mathrm{Abs}\;\mathrm{ExtB}\right)}{\left(\mathrm{Abs}\;\mathrm{PC}-\mathrm{Abs}\;\mathrm{CB}\right)}\right)*100$$

where Abs PC (Positive Control) is the absorbance of PBS with 5% EtOH, tyrosinase and L-Dopa substrate; Abs CB (Control Blank) is the absorbance of PBS with 5% EtOH and L-Dopa substrate; Abs Ext (Extract) is the absorbance of the tested invasive plant extract in the PBS buffer, tyrosinase, L-Dopa substrate and Abs ExtB (Extract Blank) is the absorbance of the tested invasive plant extract in the PBS buffer and L-Dopa substrate.

### 3.5. HPTLC-Bioautography/HRMS Analysis

VisionCATS 2.4 software monitored all the following steps and the photographs were obtained with a TLC visualizer from CAMAG (Muttenz, Switzerland). The plates were washed with MeOH to remove any impurities and dried for 2 h at 110 °C with the digital hot plate (DC).

#### 3.5.1. High Performance Thin layer chromatography (HPTLC)

Using automatic TLC Sampler 4 (ATS 4), 5 µL of crude ethanolic extracts at a concentration of 10000 µg/mL were loaded into 6 mm of length bands, with a distance of 9 mm between each band, leaving 10 mm from the bottom of the plate and 20 mm spacing from the edges. The horizontal elution over 70 mm of the polyphenolic compounds was carried out with an automatic development chamber (ADC2) at RT without humidity control. The mobile phase was composed of EtOAc:FA:AcOH:H_2_O in the proportions 100:11:11:27 (*v:v:v:v*) previously described [99] and was used with a saturation of 20 min. Chemical derivatization (Derivatizer) was performed with 2 mL of NEU reagent then 2 mL of PEG reagent. The reading was performed at 366 nm.

#### 3.5.2. HPTLC-Bioautography

Crude ethanolic extracts (5 µL) were loaded onto HPTLC and developed by using the same mobile phase and developed plates were dried at RT. The L-Dopa substrate solution at 4500 µg/mL, also containing PEG 10,000 at 7500 µg/mL and CAPS at 2500 µg/mL, was prepared in 20,000 µM phosphate buffer solution, pH 6.8 according to Chandana and Morlock [100]. Piezoelectric spraying (blue nozzle, level 6) of 1.5 mL was performed (Derivatiser) and the plate was dried for 2 min with cold stream of fan. Then 1.5 mL of tyrosinase enzymatic solution (at 400 U/mL) were sprayed. Incubation was then carried out for 15 min at RT in a humid container. Then, the plate was dried for 15 min in the open air protected from light. The plate was visualised in white light. The background of the plate assumed a grey colour and white zones of tyrosinase inhibition were observed, indicating the potential active compounds present in the extracts of *P. cuspidatum*.

#### 3.5.3. HPTLC-Bioautography/HRMS

The active areas of the tyrosinase plate were analyzed *via* a TLC/MS 2 interface with oval elution head (4 mm × 2 mm). The on-line elution of the zones was carried out with an ACN:H_2_O mixture (65:35) (*v:v*) at a flow rate of 200 µL/min and was provided using an Ultimate 3000 RSLC system. A filter was mounted between the TLC/MS interface and the ion source. Mass spectra experiments were carried out on a maXis UHR-Qq-TOF mass spectrometer (Bruker, Bremen, Germany) with an electrospray ionization (ESI), working in negative ionization mode. The pressure of the nebulizing gas was set at 0.6 bar, the flow rate and temperature of dry gas were set at 7.0 L/min and 200 °C, respectively. The capillary voltage was set at 4 kV. Full Scan MS spectra in the *m/z* 50–1650 scan range were acquired at 1 Hz. DataAnalysis 4.4 software (Bruker) was used for evaluation of the collected data.

### 3.6. UHPLC-UV/HRMS/MS Analysis

Chromatographic analyses were performed using an Ultimate 3000 RSLC system equipped with an autosampler, a binary pump, a thermostatic column compartment and a diode array detector (DAD) at 195–800 nm (Dionex, Germering, Germany). The column was a Luna Omega C18 (150 mm × 2.1 mm; 1.6 µm) (Phenomenex, Le Pecq, France). The mobile phase was composed of H_2_O (A) and ACN (B) both acidified with 0.1% of FA. Elution was performed at a flow rate of 500 µL/min and with the following binary gradient program: starting with 3% of solvent B during 0.1 min, 3–95% from 0.1 to 21 min, 95% from 21 to 24 min, 95–3% from 24 to 24.5 min. Then the column was re-equilibrated with 3% of solvent B during 3 min. The column temperature was set at 40 °C and 0.35 µL of the crude ethanolic extracts were injected. The MS/MS experiments were carried out on a maXis UHR-Qq-TOF mass spectrometer with an ESI source, working in negative ionization mode. The pressure of the nebulizing gas was set to 2 bar, the flow rate and temperature of dry gas were set at 9.0 L/min and 200 °C, respectively. The capillary voltage was set at 4 kV. The mass spectra were recorded at 2 Hz in the 50–1650 *m/z* range. All the MS data were processed using DataAnalysis 4.4 software. Auto MS/MS analyses were performed to obtain structural information in order to propose an identification for molecules using both the SmartFormula algorithm and PubChem database.

### 3.7. Statistical Analysis

Excel 2016 software (Microsoft, Redmond, WA, USA) and XLSTAT 2022 by Addinsoft were used for all the analyses. The means are represented as mean ± standard deviation (S.D.) (*n* = 3) for all the biological assays. An analysis of variance (one-factor ANOVA) followed by multiple comparison of means, using Student’s *t*-test, Dunnett’s test and Fisher’s F-test (LSD), was implemented in order to statistically compare the data obtained. The samples were compared with the positive control and/or between them considering the same enzyme. Two samples were considered significantly different at *p* < 0.05.

## 4. Conclusions

The present work proposes a reliable strategy using powerful analytical techniques for the screening and evaluation of the dermo-cosmetic potential of the aerial parts (AP) and root parts (RP) of *Polygonum cuspidatum*, an invasive plant. Antioxidant, anti-tyrosinase and, for the first time on this species, anti-hyaluronidase, anti-elastase and anti-collagenase capacities were examined. The results revealed strong antioxidant (DPPH and equivalent SOD activity using an indirect assay of XOD) and anti-collagenase activities, moderate anti-hyaluronidase activity, while weak anti-elastase and anti-tyrosinase activities were observed for the crude ethanolic extracts. The main specialized metabolites of the AP and RP identified through UHPLC/HRMS/MS, in correlation with previous studies, were stilbenes, quinones, flavonoids and phenolic acids. Different standards (resveratrol, polydatin, emodin and chlorogenic acid) selected and screened on the same enzymatic targets made it possible to correlate observed residual activities of the AP and RP extracts of *P. cuspidatum* from Savoie Mont Blanc and their chemical compositions.

A structure–activity study was thus conducted on the main molecular families, widely represented in the genus *Polygonum*. We demonstrated that the whole invasive plant could be used (AP, RP and AP:RP), representing a real material resource for the Savoie Mont Blanc territory and a possible new alternative treatment for sustainable management. Both AP and RP are good antioxidant, anti-hyaluronidase and anti-elastase candidates. AP have shown a very good collagenase inhibition property while RP is a better tyrosinase inhibitor than AP. Further in vivo studies have to be performed in order to confirm the in vitro results and evaluate the potent anti-aging and whitening activity of both AP and RP of *P. cuspidatum*. Finally, a new alternative way for the valorization of invasive plants in the dermo-cosmetic sector was proposed and could be used for other problematic invasive plant species.

## Data Availability

All data is comprised in the manuscript.

## Supplementary Materials

The following supporting information can be downloaded at: https://www.mdpi.com/article/10.3390/plants12010083/s1, Figure S1. Evaluation of DPPH antioxidant capacities of AP and RP extracts of *P. cuspidatum* from Savoie Mont Blanc. *n* = 3 independent experiments for each concentration (µg/mL). Bars depict ± S.D.; Figure S2. Evaluation of elastase (a), collagenase (b) and tyrosinase (c) residual activities of ethanolic extracts of mixture (AP:RP) of invasive *P. cuspidatum*. PC = Positive Control; (A) = N-Methoxysuccinyl-Ala-Ala-Pro-Val-chloromethyl ketone; (B) = 1,10-Phenanthroline, monohydrate and (Gla) = glabridin. *n* = 3 independent experiments for each concentration (µg/mL). Bars depict ± S.D.; Figure S3. Electropherograms obtained with CE-UV for hyaluronidase assays: control assay in absence of inhibitor standards (I) and the effect at 50 µg/mL of chlorogenic acid (II) and resveratrol (III). Hyal: hyaluronidase and HA: hyaluronic acid. Reaction mixture in IB of control: 200 µg/mL Hyal and 800 µg/mL of HA. Modulation of hyaluronidase activity experimental conditions: 200 µg/mL of Hyal, 800 µg/mL of HA and 10 µg/mL of filtered crude extract. Incubation at 37 °C for 180 min. IB: 164 µg/mL sodium acetate (pH 4.3). Electrophoretic separation conditions: BGE: 3854 µg/mL, ammonium acetate (pH 8.9); anodic injection: 1.5 psi for 5 s; (14 nL); separation: +15 kV at 25 °C; detection: λ = 200 nm; rinse between analyses at 30 psi: 5 min NaOH (1 M), 0.5 min water and 3 min BGE; bare-silica capillary: 0.57 m total length, 0.47 m detection length, 50 μm i.d. Peaks identification: electroosmotic flow (EOF) at 6 min and peak (A): tetrasaccharide migrating at 13 min.; Table S1. Putative identification of main compounds presents in AP and RP of invasive *P. cuspidatum* from Savoie Mont Blanc, using UHPLC/HRMS/MS.; Supplementary Materials and methods.
