# Supplementary material for: Screening and Evaluation of Dermo-Cosmetic Activities of the Invasive Plant Species Polygonum cuspidatum
Source: Plants (Basel). 2022 Dec 23;12(1):83. doi: 10.3390/plants12010083 (PMC9823685; doi:10.3390/plants12010083)
Supplement: Supplementary file 1 [file plants-12-00083-s001.zip › plants-2050832-supplementary.pdf]

plants

Supplementary Materials

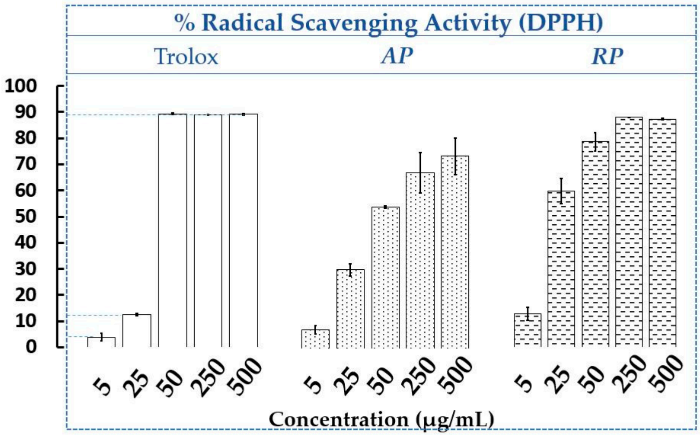

Figure S1. Evaluation of DPPH antioxidant capacities of AP and RP extracts of *P. cuspidatum* from Savoie Mont Blanc. n = 3 independent experiments for each concentration (µg/mL). Bars depict ± S.D.

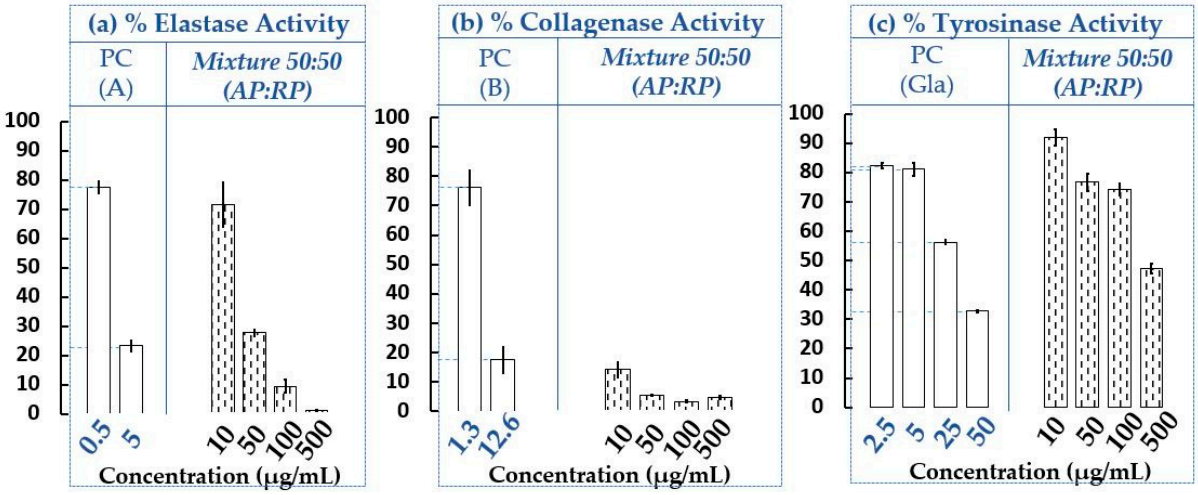

Figure S2. Evaluation of elastase (a), collagenase (b) and tyrosinase (c) residual activities of ethanolic extracts of mixture (AP:RP) of invasive *P. cuspidatum*. PC = Positive Control; (A) = N-Methoxy-succinyl-Ala-Ala-Pro-Val-chloromethyl ketone; (B) = 1,10-Phenanthroline, monohydrate and (Gla) = glabridin. n = 3 independent experiments for each concentration (µg/mL). Bars depict ± S.D.

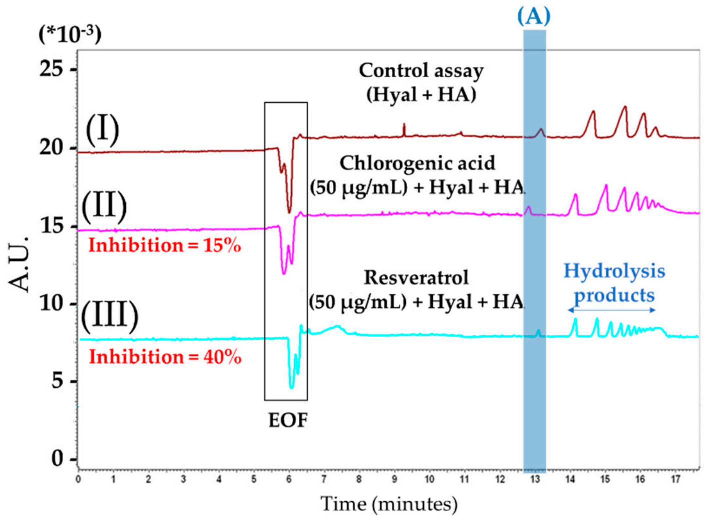

Figure S3. Electropherograms obtained with CE-UV for hyaluronidase assays: control assay in absence of inhibitor standards (I) and the effect at 50 µg/mL of chlorogenic acid (II) and resveratrol (III). Hyal: hyaluronidase and HA: hyaluronic acid. Reaction mixture in IB of control: 200 µg/mL Hyal and 800 µg/mL of HA. Modulation of hyaluronidase activity experimental conditions: 200 µg/mL of Hyal, 800 µg/mL of HA and 10 µg/mL of filtered crude extract. Incubation at 37°C for 180 min. IB: 164 µg/mL sodium acetate (pH 4.3). Electrophoretic separation conditions: BGE: 3 854 µg/mL, ammonium acetate (pH 8.9); anodic injection: 1.5 psi for 5 s; (14 nL); separation: +15 kV at 25°C; detection: λ = 200 nm; rinse between analyses at 30 psi: 5 min NaOH (1 M), 0.5 min water and 3 min BGE; bare-silica capillary: 0.57 m total length, 0.47 m detection length, 50 µm i.d. Peaks identification: electroosmotic flow (EOF) at 6 min and peak (A): tetrasaccharide migrating at 13 min.

Table S1. Putative identification of main compounds present in AP and RP of invasive *P. cuspidatum* from Savoie Mont Blanc, using UHPLC/HRMS/MS.

Part of *P. cuspidatum*	Molecular Family	Retention time (min)	MS $[M-H]^-$ molecular ion	MS/MS fragments	Identification of compounds[1]
Aerial Part (AP)	Phenolic acids	3.71	353.0888	191/179	3-*O*-Caffeoylquinic acid
		4.35	337.0939	191/163	*p*-coumaroylquinic acid
		4.59	353.0883	191	5-*O*-caffeoylquinic acid (1)
	Flavan-3-ol	4.60	289.0727		Catechin
	Stilbenes	6.45	389.1245	227	Polydatin (2)
		8.25	227.0727		Resveratrol (3)
	Flavonols	7.10	433.0784	301	Quercetin 3-*O*-pentoside
		7.26	447.0950	301	Quercitrin
	Quinones	8.91	431.0985	269	Emodin-8-*O*-*D*-glucoside
		9.39	517.0990	269	13 emodin-8-*O*-(6′-*O*-malonyl)-glucoside
		13.87	269.0463		Emodin (4)
Root Part (RP)	Phenolic acid	4.59	353.0878	191	5-*O*-caffeoylquinic acid (1)
	Flavan-3-ols	4.60	289.0725		Catechin
		4.97	577.1359	289	Procyanidin dimer B
		5.23	289.0727		Epicatechin
	Stilbenes	5.69	405.1206	243	Piceatannol glucoside
		5.78	389.1263	227	Resveratroloside
		6.45	389.1258	227	Polydatin (2)
		6.96	541.1359	389/227	Polydatin gallate
		8.24	227.0728		Resveratrol (3)
	Flavonol	7.26	447.0942	301	Quercitrin
	Quinones	8.92	431.1003	269	Emodin-8-*O*-*D*-glucoside
		9.37	517.1001	269	13 emodin-8-*O*-(6′-*O*-malonyl)-glucoside
		9.89	445.1156	283	Physcion glucoside
		10.52	283.0626		Physcion
	Naphthols	8.83	407.1368	245	Torachrysone glucoside
		13.99	245.0834		Torachrysone

[1]Identifications by comparison of MS spectra data with the literature and their identification were tentative. (1) 5-*O*-caffeoylquinic acid = chlorogenic acid; (2) Polydatin; (3) Resveratrol and (4) Emodin: compounds of interest on the four enzyme targets studied (hyaluronidase, elastase, collagenase, tyrosinase).

Supplementary Materials and methods

Extraction

A mixture 50:50 (*v:v*) was made from the two extracts of aerial and root parts (AP:RP) allowing to obtain a solution at 5 000 µg/mL. It was then diluted in cascade to obtain solutions at 200; 1 000 and 2 000 µg/mL used in enzymatic tests.

DPPH assay

The DPPH radical scavenging assay was used to determine antioxidant activity using the stable free radical 2,2-diphenyl-1-picrylhydrazyl and trolox was used as the reference standard. A 200 µM DPPH solution (equivalent to 79 µg/mL) was prepared in EtOH. A trolox solution was prepared in EtOH at 10 000 µg/mL then diluted in cascade to obtain solutions at 5 000; 1 000; 500 and 100 µg/mL. In each well, 10 µL of extract (prepared according to 3.3.) or trolox were mixed with 190 µL of DPPH reagent. The plate was incubated for 30 min at RT in the dark then agitated. The absorbance (Abs) reading (λ = 516 nm) was performed using a CLARIOstar Plus microplate reader. Experiments were performed in triplicate. The percentage of radical scavenging activity was calculated using the following equation:

$$\text{Inhibition (\%)} = \left(\frac{\text{Abs CB} - \text{Abs Sample}}{\text{Abs CB}}\right) * 100$$

where Abs CB (Control Blank) is the absorbance of EtOH and reagent; Abs Sample is the absorbance of the tested invasive plant extract and reagent or reference trolox.

Elastase assay

For the mixture 50:50 extract at 5 000 µg/mL, 20 µL of sample were added to 30 µL of reaction buffer and 100 µL of elastase to obtain the same final concentration (500 µg/mL) as the extracts of AP and RP tested separately. The rest of the operating protocol was identical.

Collagenase assay

For the mixture 50:50 extract at 5 000 µg/mL, 20 µL of sample was added to 60 µL of reaction buffer and 100 µL of collagenase to obtain the same final concentration (500 µg/mL) as the extracts of AP and RP tested separately. The rest of the operating protocol was identical.

Tyrosinase assay

For the mixture 50:50 extract at 5 000 µg/mL, 20 µL of sample were added to 20 µL of PBS and 40 µL of tyrosinase solution to obtain the same final concentration (500 µg/mL) as the extracts of AP and RP tested separately. The rest of the operating protocol was identical.
